# Comparing the Effects of Different Smoothing Algorithms on the Assessment of Dimensionality of Ordered Categorical Items with Parallel Analysis

**DOI:** 10.1371/journal.pone.0148143

**Published:** 2016-02-04

**Authors:** Rudolf Debelak, Ulrich S. Tran

**Affiliations:** 1 SCHUHFRIED GmbH, Mödling, Austria; 2 University of Zurich, Zurich, Switzerland; 3 University of Vienna, Vienna, Austria; Qom University, ISLAMIC REPUBLIC OF IRAN

## Abstract

The analysis of polychoric correlations via principal component analysis and exploratory factor analysis are well-known approaches to determine the dimensionality of ordered categorical items. However, the application of these approaches has been considered as critical due to the possible indefiniteness of the polychoric correlation matrix. A possible solution to this problem is the application of smoothing algorithms. This study compared the effects of three smoothing algorithms, based on the Frobenius norm, the adaption of the eigenvalues and eigenvectors, and on minimum-trace factor analysis, on the accuracy of various variations of parallel analysis by the means of a simulation study. We simulated different datasets which varied with respect to the size of the respondent sample, the size of the item set, the underlying factor model, the skewness of the response distributions and the number of response categories in each item. We found that a parallel analysis and principal component analysis of smoothed polychoric and Pearson correlations led to the most accurate results in detecting the number of major factors in simulated datasets when compared to the other methods we investigated. Of the methods used for smoothing polychoric correlation matrices, we recommend the algorithm based on minimum trace factor analysis.

## Introduction

The assessment of dimensionality of a set of variables is a central issue in psychological and educational measurement, closely connected to theory building and psychological scale construction [1,2]. Very common exploratory approaches for determining the dimensionality of a set of variables are based on exploratory factor analysis (EFA) and principal component analysis (PCA), see [3]. Since an incorrect assessment of the dimensionality underlying a set of variables can be of severe consequences for the interpretation of empirical data [4,5], multiple formal criteria have been developed to determine the number of factors or components to retain. For PCA, researchers have proposed, among other methods, the eigenvalue-greater-than-one criterion [6], parallel analysis [7,8], the scree test [9], and Velicer’s Minimum Average Partial (MAP) rule [10]. In the context of EFA, an adaptation of parallel analysis [11], and, for maximum likelihood factor analysis, chi square significance tests and approaches based on information criteria like the Akaike Information Criterion [12] and the Bayesian Information Criterion [13] are among the methods which were suggested to assess the dimensionality of an item set. Among these methods, the adaptations of parallel analysis (PA) are commonly regarded to provide an accurate assessment under many conditions [2], although it has been noted that there is less evidence for the accuracy of PA in the context of EFA [14].

In applications of PA to discrete ordinal variables, Pearson correlations are often used, based on the assumption that a linear model underlies the variables. However, this approach has been shown to lead to biased results under specific conditions. The use of Pearson correlations may lead to biased results in binary data [15], and the application of tetrachoric correlations is recommended in this case. A more recent paper provided additional evidence that an EFA or PCA based on Pearson correlations may lead to biased results when compared to approaches based on tetrachoric or polychoric correlations, especially when response distributions of the analyzed variables are skewed [16]. This result is in line with theoretical observations, since it has been remarked by multiple authors that the possible range for Pearson correlation coefficients between ordered categorical variables is influenced by the relative frequency of the categories and may be limited in skewed variables, while the variables’ skewness has no comparable effect on the estimations for tetrachoric and polychoric correlation coefficients [17,18,19].

Some writers thus suggested the application of PCA or EFA based on tetrachoric or polychoric correlations in ordinal variables as an alternative approach [2,15]. The use of polychoric and tetrachoric correlation coefficients assumes that a normally distributed latent variable underlies each of the observed ordinal variables, an assumption which may, however, be violated in specific applications [18].

Multiple studies have been carried out to compare the accuracy of methods on the basis of polychoric correlation matrices with that of methods based on Pearson correlations. An example is [20], which compared maximum likelihood estimation based on Pearson correlations with weighted least squares mean and variance adjusted (WLSMV) estimation based on polychoric correlations in the context of confirmatory factor analysis. In this simulation study, WLSMV estimation led to more accurate results in the analysis of categorical variables, especially when the number of categories was small. A similar argument against the appropriateness of maximum likelihood estimation in the analysis of categorical data has been made by Olsson [21], again based on the results of a simulation study.

However, an often reported objection to the use of polychoric correlation matrices is the presence of indefinite correlation matrices [2,22]. As has been discussed by numerous authors (see, e.g., [23] and [19], p. 349) indefinite correlation matrices may be problematic for specific methods of multivariate analysis, like factor analysis. For this reason, several authors have advised against the use of tetrachoric and polychoric correlation matrices in factor analysis or described non-convergence as a severe problem [2,17]. Although specific variants of factor analysis have been proposed which may be applied to indefinite correlation matrices [24,25], the presence of negative eigenvalues forbids interpreting eigenvalues with regard to the variance that is explained by the respective factors [16]. Several authors [2,15,26] have thus suggested to apply smoothing algorithms [27] which transform indefinite correlation matrices into positive definite ones, to avoid this problem. However, only few studies have investigated the effect of applying smoothing algorithms on dimensionality assessment with PCA or EFA in ordered categorical variables [2,16,26], and results were also mixed. While Garrido et al. [16] recommended the use of smoothed polychoric correlations to assess the dimensionality of sets of ordinal items, Timmerman and Lorenzo-Seva [2] did not generally advise their use, because of computational problems, like convergence problems in the calculation of the polychoric matrices. The study of Debelak and Tran [26] indicated that the application of different smoothing algorithms may lead to slightly different results in the special case of a PCA of binary items which they investigated. Based on these results, little recommendations regarding the choice of an appropriate smoothing algorithm can be given to the practical researcher who wishes to apply PA to assess the dimensionality of ordinal variables. Thus, the present study examines the effect of various smoothing algorithms on dimensionality assessment with PCA or EFA in ordered categorical variables by the means of a simulation study.

This paper is organized as follows. In the next section, previous studies on the application of PA in EFA and PCA with and without smoothing algorithms to ordinal data are summarized. Subsequently, we discuss several smoothing algorithms for indefinite correlation matrices described in the literature. We then present the results of a simulation study on the application of a PCA and principal axes factoring of smoothed polychoric and Pearson correlations. A summary of our findings and recommendations for applied researchers are provided in the Discussion section.

### The use of parallel analysis with ordinal data

Many authors recommended PA, a method which was suggested by Horn [8] in the context of PCA, to determine the number of factors or components in EFA or PCA [4,28]. In its original form, PA consists of two major steps: First, the eigenvalues of the correlation matrix of the dataset are computed. In a second step, a number of datasets which are comparable to the original dataset with respect to the sample size, the number of items and the distribution of the responses, but consist of uncorrelated items, are simulated. Based on these simulated datasets, the distribution of random eigenvalues is obtained. The number of dimensions underlying the dataset is determined by comparing the eigenvalues observed in the dataset and the distribution of random eigenvalues which are observed when there is no relationship between the items. An alternative method for conducting PA in EFA, which was based on principal axis factoring (PAFA), has been described by [11] and [29]. In the alternative approach, a modified correlation matrix is used, which contains communality estimates in its diagonal. In the simulation study presented by Timmerman and Lorenzo-Seva [2], a comparison of this modified approach to the original PA of Horn [8] indicated that the original approach led to more accurate results. This study also investigated the accuracy of PA with minimum rank factor analysis (MRFA). This approach for EFA aims at minimizing the amount of unexplained variance given a predefined number of common factors under the constraint that the reduced correlation matrix is positive-definite [2]. It was found that PA, when applied with MRFA, led to slightly more accurate results than PA applied to PCA in the datasets of their simulation study. In this study, the latent factors underlying all datasets were uncorrelated.

Timmerman and Lorenzo-Seva [2] also compared the accuracy of PA when applied to a PCA and EFA of polychoric and Pearson correlations. They found that approaches based on polychoric correlations generally led to accurate results, but also reported problems with non-convergence of the polychoric correlation matrices in the calculation of critical eigenvalues. In their study, PA was regarded non-convergent if 25 simulated matrices in a row were found to be indefinite or did not converge. In all cases where the polychoric correlation matrices converged, PCA and MRFA based on polychoric correlation matrices was found to be more accurate than PCA and MRFA based on Pearson matrices. Nevertheless, Timmerman and Lorenzo-Seva [2] concluded that the convergence problem prohibits the general application of PCA of polychoric correlations to empirical datasets. For Pearson correlations, PCA led to slightly more accurate results using the 95^th^ or 99^th^ percentile of the random eigenvalue distributions [7] than their mean [8] in datasets with uncorrelated latent factors. The simulation study of Timmerman and Lorenzo-Seva [2] calculated polychoric correlations using the two-step algorithm of Olsson [30]. This algorithm is based on maximum likelihood estimation of the correlation coefficient, while the thresholds which define the relation between the latent continuous variables and the categories of the manifest ordinal variable are calculated directly from the observed cumulative marginal proportions of each category in the data (see also the Methods section).

A recent simulation study [16] further investigated the accuracy of PA applied to PCA and MRFA in assessing the dimensionality of ordinal variables. Results indicated that PA with Pearson correlations is inaccurate with large levels of skewness and the use of PA with polychoric correlations was recommended for the dimensionality assessment of ordinal data. PCA tended to lead to more accurate solutions than MRFA in datasets with few (four) variables per factors, and also in datasets with correlated latent factors, whereas it was less accurate in datasets with a medium (eight) or large (12) number of variables per factor. Garrido et al. [16] used a smoothing algorithm described by Knol and Berger [31] (see next section) to circumvent the problem of indefinite polychoric correlation matrices.

Some further improvements on the original PA approach have been suggested as well, e.g., [7,32]. While Horn [8] originally recommended the use of the mean of the distribution of the simulated eigenvalues in PA to determine the number of components to retain, Glorfeld [7] first advocated using the 95^th^ percentile as a threshold to determine the number of components. This leads to more accurate results in datasets with uncorrelated factors, while Horn’s original method seems to lead to more accurate results when factors are correlated [16,33,34].

Further, several authors criticized that the procedure used in traditional parallel analysis is based on a model with 0 underlying factors [32,35]. It has been argued that, while this procedure may be appropriate to assess the presence of a single common factor in the data, it may be inappropriate for evaluating the presence of additional factors. Green et al. [32] thus have suggested incorporating all precedent k factors or components in modeling the reference distribution of the subsequent (k+1)th eigenvalue to improve the accuracy of parallel analysis. The (k+1)th critical eigenvalue is obtained by fitting a factor model with k factors on the dataset. In a second step, new datasets with the same number of variables and subjects as in the original dataset are simulated based on a model with k factors, whose loadings are identical to those observed in the original dataset. A descriptive statistic (like the mean or a specific percentile value) is calculated to summarize the distribution of the (k+1)th eigenvalue in these simulated datasets. This descriptive statistic is further used as a critical threshold for the (k+1)th eigenvalue. This method has been further investigated in a row of recent studies [36,37].

Although it has been argued that PA is no exact mathematical procedure [2,38], it has been found to provide an accurate assessment of the dimensionality of an item set under many conditions. Zwick and Velicer [5] evaluated the performance of PA in a simulation study, varying the size of the simulated item and respondent sample, the number of major components, and the magnitude of loading of the variables on each major component. They found that PA led generally to the most accurate results compared to the eigenvalue-greater-than-one criterion [6], the scree test [9], and Velicer’s minimum average partial method [10]. Humphreys and Montanelli [29] evaluated PA in EFA by applying it to simulated datasets which were created from predefined correlation matrices. They found that PA led to consistent results which corresponded to the number of dimensions defined by the correlation matrices. Peres-Neto, Jackson and Somers [39] compared the performance of PA with other stopping rules in PCA under several conditions, including different correlation matrices and different sizes of samples and variables. They found PA to be among the most accurate methods to determine the dimensionality of a test.

### Some smoothing procedures for indefinite correlation matrices

The application of polychoric correlation matrices in the analysis of discrete ordinal variables assumes that a latent continuous normally distributed variable underlies each observed variable. The polychoric correlation is a maximum likelihood estimator of the Pearson correlation between the two normally distributed underlying variables [19]. However, since the estimation of the polychoric correlation matrix is based on different marginals for each correlation coefficient, the resulting matrix may be indefinite [31].

In order to apply PCA and factor analytical methods in these cases, several smoothing algorithms have been suggested, e.g., [2,16]. We will describe some selected algorithms below. All described smoothing algorithms have in common that they do not alter positive definite correlation matrices to which they are applied.

Higham [40] suggested an algorithm which searches for the symmetric positive definite matrix X which minimized the Frobenius norm to a given symmetrical matrix A. The Frobenius norm is defined by $\|A-{X\|}_{F}=\sum_{i,j}{\left(a-x\right)}_{i,j}^{2}$. This algorithm has been implemented in the R package Matrix [41].

An alternative approach for transforming indefinite correlation matrices into positive definite ones was described by Bentler and Yuan [42]. Given a symmetric indefinite matrix *R*, [42] proved that a positive definite matrix R* can be obtained by

$$R^{*}=\Delta R_{0}\Delta +D_{R}$$ (1)

In formula (1), *R*_0_ results from *R* by setting its diagonal entries to 0, *D*_*R*_ is the diagonal matrix containing the diagonal elements of *R*. Furthermore, Δ is a positive definite matrix which meets the condition that *D*_*R*_ − Δ^2^(*R*−*D*) is positive definite, where *D* is a diagonal matrix with nonzero elements such that (*R*−*D*) is positive semidefinite. Bentler and Yuan [42] further demonstrate a variation of their approach which is based on minimum trace factor analysis [43]. This approach can be applied using functions implemented in the R packages psych [44] and Rcsdp [45], see [26].

Knol and Berger [31] described another smoothing algorithm, which was later also suggested by [15] and applied in the simulation studies [16] and [26]. This smoothing algorithm is based on the idea to set eigenvalues which are smaller than a predefined threshold δ and to correct the associated eigenvectors accordingly to obtain a correlation matrix whose eigenvalues are thus greater than or equal to δ. Let *R* = *KDK*^t^ be the eigendecomposition of the symmetric matrix *R*, with *D* being a diagonal matrix of its eigenvalues *λ*_*i*_, *K* the matrix of the corresponding normalized eigenvectors, and *D*^+^ a diagonal matrix in which all eigenvalues lower than δ have been replaced with δ, see [31]. Then, if *Diag*(*X*) denotes the matrix containing the entries of the main diagonal of *X*, a correlation matrix with eigenvalues greater than δ can be obtained by calculating. *R*(*δ*) = [*Diag*(*KD*^+^*K*^*t*^)]^−1/2^*KD*^+^*K*^*t*^[*Diag*(*KD*^+^*K*^*t*^)]^−1/2^. Debelak and Tran [26] reported that this smoothing algorithm leads to results similar to those obtained with the algorithm of Higham [40] when used in PCA of binary items.

Another approach for smoothing indefinite correlation matrices has been proposed by Knol and ten Berge [23]. In contrast to the smoothing algorithms by Bentler and Yuan [42], Higham [40], and Knol and Berger [31], this smoothing algorithm allows defining rows and columns of the original correlation matrix which should not be altered by the smoothing algorithm. For the remaining parts of the correlation matrix *R*, an approximation matrix R* is determined which is positive definite, symmetrical with *Diag*(R*) = *I* and additionally meets the criterion that the trace of (*R* − R*)^2^ is minimized. For finding this minimum, an iterative algorithm based on [43] is proposed. Although the Knol and ten Berge algorithm [23] is interesting for many practical applications, it is, to our knowledge, currently not implemented in any widely available software program.

Additional smoothing algorithms have been made available in specific software programs and packages. For the users of the R programing language, appropriate functions are contained in several software packages, like sfsmisc [46] and psych [44]. However, these algorithms are based on similar ideas as the ones described above, and are therefore not described in further detail.

It should be noted that the approaches described by Bentler and Yuan [42] and Knol and Berger [31] generally alter only few cells in an indefinite correlation matrix to obtain a positive definite correlation matrix, which constitutes an important difference to the algorithm of Higham [40] that makes small modifications to the complete correlation matrix. In contrast to this algorithm, the method of Bentler and Yuan [42] is based on the idea of rescaling variables of the indefinite correlation matrix which correspond to Heywood cases. In their simulation study on binary variables, Debelak and Tran [26] found that the algorithm of Bentler and Yuan [42] led to more accurate results than the algorithm of Higham [40] under most examined conditions.

### Goals of the current study

A number of studies compared the accuracy of PA in assessing the dimensionality of an item set in the context of PCA and EFA when applied to polychoric correlation matrices with its accuracy when applied to Pearson correlation matrices [2,15,16,26]. These studies generally found that PA based on polychoric matrices led to an accurate assessment of the dimensionality of an item set, but the problem of indefinite polychoric correlation matrices led to the recommendation to use smoothing algorithms [15], and to the evaluation of various smoothing algorithms [2,16, 26]. Although the application of smoothing algorithms has been shown to enhance the accuracy of PA of polychoric correlations, little is known on the effects of different smoothing algorithms on the accuracy of PA. Although this issue has been examined in [26], this study was constrained to the investigation of PCA applied to tetrachoric correlations, so the generalization of these results to more general conditions (i.e., items with three or more response categories) or to the application of EFA remains unclear. The main goal of the current study was therefore to evaluate the application of three different widely available smoothing algorithms, and their effect on the accuracy of Horn’s [8] and Glorfeld’s [7] variations of PA in the context of PCA and principal axes factor analysis (PAFA) ([11,29]) as a method of EFA. It should be noted that several recent simulation studies [2,16] included MRFA as a promising method of EFA. This method was not included in this simulation study, since it is, to the best of our knowledge, currently not available in published R packages.

In our study, we restricted the application of smoothing algorithms to the three algorithms described by Bentler and Yuan [42], Higham [40] and Knol and Berger [31]. This choice was motivated by a number of considerations: First, these smoothing algorithms are available in the open source software R [47] and can therefore be easily applied in practical research. Second, these smoothing algorithms are based on specific, but different criteria for the smoothing method, and lead to different positive definite matrices when applied to an indefinite matrix; they can therefore not be regarded as interchangeable. Third, these smoothing algorithms tend to be computationally fast, which makes them attractive in practical application. The algorithm of Knol and ten Berge [23] was not included in our study, since it is, to our best of knowledge, currently not implemented in any available software package and seems to be hardly used in practical research. Since it is also possible to apply PCA and PAFA to indefinite correlation matrices, we further evaluated the accuracy of PA if no smoothing algorithms are used in the application of PA on polychoric correlation matrices.

## Methods

We carried out a simulation study to compare the accuracy of PA with PCA and PAFA based on polychoric correlations, with and without applying a smoothing algorithm, with the results obtained from a PCA and PAFA based on Pearson correlations. In our study, responses from a person sample to an item set were simulated based on several specific factor models.

The simulated datasets varied in the following aspects: (a) the size of the respondent sample, (b) the size of the item set, (c) the number of response categories, (d) the skewness of the response distribution, (e) the underlying factor model, and (f) the applied correlation method and smoothing algorithm. The design of our simulation study resembles those of previous studies on the accuracy of PA reported in the literature, e.g., [2,15,26,33]. Under each condition, the critical eigenvalue for determining the dimensionality of the item set was obtained as the mean (Horn [8]) or the 95^th^ percentile (Glorfeld [7]) of the random eigenvalue distributions, respectively. When parallel analysis was applied, the random eigenvalue distribution was obtained by drawing 100 random samples from a simulated response matrix under each condition. Under each condition, the random samples were generated through a permutation of the original response matrix.

In the context of PAFA, the approach of [11], in which the correlation matrix is modified to contain communality estimates in its diagonal, was further used to apply PA. In the cases where this approach was combined with a smoothing procedure, the smoothing procedure was first applied to obtain a positive definite correlation matrix. In a second step, the Humphreys and Ilgen approach [11] was then used to determine the dimensionality of the variable set.

### Size of the respondent sample

Three different sizes of samples were used, which consisted of 200, 500 and 1000 respondents. This choice aimed to reflect the sample sizes found in practical applications of PCA and EFA. Numerous studies have already demonstrated that the size of the respondent sample has a significant impact on the accuracy of PA [2,16,26,33]. We therefore expected to obtain more accurate results in datasets with a greater respondent sample.

### Underlying factor model

The response matrices analyzed in our study were based on a factor model which contained major, minor, and unique factors. Following earlier studies [2,48], the inclusion of many minor factors in our simulations aimed at simulating the effect of random factors on the observed variables which go beyond the effects of unique factors. These minor factors can be thought of as representing random influences on the observed variables and noise which cannot be controlled in applied research. Similar to the simulation study of Timmerman and Lorenzo-Seva [2], the population correlation matrix ∑_*pop*_ of the latent variables underlying the discrete responses to the items was given as

$$\Sigma_{pop}=w_{ma}\Lambda_{ma}\Lambda_{ma}^{t}+w_{mi}\Lambda_{mi}\Lambda_{mi}^{t}+w_{un}I_{J}$$ (2)

In this formula, Λ_*ma*_ and Λ_*mi*_ stand for the factor loading matrices of the major and minor factors, respectively, with $\Lambda_{ma}^{t}$ and $\Lambda_{mi}^{t}$ being the transposed loading matrices. In our simulations, each major factor loaded on a constant number of variables, which was 5 or 15, depending on the simulated condition. Since we simulated conditions with one or three major factors, cases with 5, 15 or 45 variables were simulated. If a major factor loaded on a specific variable, the specific loading Λ_*ma*_ was set to 1, otherwise it was set to 0. The simulated factor models can be thus interpreted as having a simple structure with uncorrelated factors.Λ_*mi*_ contained the loadings of minor factors, which were included in some of our simulations. If minor factors were present, their number was set to be 40. The loadings of the minor factors on the variables were drawn from an uniform distribution in the interval [-1;1]. *I*_*J*_ is an identity matrix of size J, with J being the number of the variables. *w*_*ma*_, *w*_*mi*_ and *w*_*un*_ are weights which determined the loadings of major and minor factors in the simulated dataset. The analyzed datasets differed with regard to the size of the loadings of the underlying factors on the observed variables. In conditions with high and medium factor loadings, *w*_*ma*_ was set to be 0.5 and 0.3 respectively. *w*_*mi*_ was set to 0 in simulated datasets where minor factors were absent, and to $\frac{0.2w_{ma}}{E(\wedge_{mi}\wedge_{mi}^{t})}$ where minor factors were present in the data. *w*_*un*_ was set to (1 − *w*_*mi*_ − *w*_*ma*_). It follows thus that in datasets without minor factors about 50% and 30% of the variance of the observed variables could be explained by the major factors in conditions with high and medium factor loadings, respectively. The resulting relationship between all major factors and the observed variables allowed us to use the number of major factors as criterion for the dimensionality of each dataset. Based on the population correlation matrix ∑_*pop*_ a set of 5, 15 or 45 continuous variables was defined which followed a multivariate standard normal distribution. This set was further transformed into a set of discrete ordinal variables by applying thresholds, which further defined the number of the response categories and the skewness of the response distribution.

### Number of categories

In our simulation study, items with two, three or four response categories were simulated. While we expected the differences between methods based on Pearson and polychoric correlations to be most pronounced in conditions with two response categories, items with three or more response categories are regularly used in psychological and clinical assessments.

### Skewness of the response distribution

For each number of response categories, a symmetrical and a skewed distribution of the observed variables were simulated. In case of a symmetrical distribution, the expected frequencies of observations were set to be equal for all response categories. In the skewed response distributions, the thresholds for defining the ordinal variables were set so that the response categories were observed with expected relative frequencies of [0.2; 0.8], [0.1; 0.2; 0.7], and [0.1; 0.1; 0.1; 0.7] under the conditions of simulated variables with two, three or four response categories, respectively. This led to response distributions with an estimated skewness of about 1.4, which may be interpreted as a meaningful departure from normality, see [16]. We expected the difference between methods based on Pearson and polychoric correlation matrices to be more pronounced under skewed response distributions.

In summary, 2 (number of major factors) x 2 (number of variables per major factor) x 3 (number of simulated respondents) x 3 (number of the response categories) x 2 (skewness of the response distribution) x 2 (presence of minor factors) x 2 (factor loadings) = 288 different conditions were simulated. Under each condition, the datasets were analyzed using four different variations of PA, which were based on PCA or EFA and used the criterion of Horn [8] or Glorfeld [7], respectively. 100 response matrices were simulated under each condition. In each simulated dataset, it was assessed how many factors or components were retained, and whether this number was identical to the number of major factors. The code used in this simulation study can be provided by the first author.

Calculation of the polychoric correlation matrices was based on algorithms implemented in the R package psych [44]. In all cases we used a two-step procedure which is based on the algorithm of Olsson [30], using item pair-specific estimators of the threshold coefficients for estimating the polychoric correlation matrix. Based on the results of a preliminary study, this approach appeared to be numerically more stable than an alternative procedure which estimated the threshold coefficients simultaneously for the whole matrix. For implementing the smoothing algorithms of Higham [40] and Bentler and Yuan [42], methods from the R packages Matrix [41], psych [44] and Rcsdp [45] were used. In order to apply the smoothing algorithm of Knol and Berger [31], code was written in R, which can be obtained from the authors. In our simulations, the constant δ was set to 0.1. We further investigated the accuracy of each parallel analysis method if no smoothing algorithm was applied.

## Results

### Overall results

We first present some tables which summarize the accuracy of the dimensionality assessment of each method under each condition. Each dimensionality assessment was considered as accurate if the number of major factors was correctly measured. In general, all smoothing algorithms led to comparable results across all simulated conditions. Therefore, only the results obtained with the algorithms of Bentler and Yuan [42] and Knol and Berger [31] will be presented in detail. Overall, these two algorithms led to the most accurate assessments of dimensionality, with the algorithm of Bentler and Yuan [42] correctly identifying the number of greater factors in about 91.5% of the simulated datasets and the algorithm of Knol and Berger [31] in about 90.7% of the simulated datasets. The algorithm of Higham [40] (90.3% correct assessments) and the analysis of the indefinite correlation matrices (89.14% correct assessments) led to only slightly less accurate results. The methods based on the analysis of Pearson correlation matrices led to a correct assessment of dimensionality in 91.3% of the simulated datasets. We now present several tables which present the percentage of simulated datasets in which the number of major factors was reported correctly by the application of parallel analysis. The entries in the individual cells of these tables are based on 3600 datasets (in columns which present results for different numbers of major factors or different numbers of variables per factor) or 2400 datasets (in columns which present results for different numbers of respondents or different response categories). The confidence intervals for the individual entries in these tables are smaller than ± 1.6 for entries based on 3600 datasets and smaller than ± 2 for entries based on 2400 datasets, based on a significance level of 0.05. However, it should be noted that the size of the confidence interval is smaller for entries closer to 0 and 100. For entries above 90, the respective confidence intervals are smaller than ± 1.0 for entries based on 3600 datasets and smaller than ± 1.2 for entries based on 2400 datasets, again based on a significance level of 0.05. Additional detailed results for the accuracy of parallel analysis in datasets with high and medium factor loadings are provided in S1 Appendix.

Table 1 summarizes the results of the simulations where the response distribution was symmetrical, with no minor factors present.

**Table 1:** Rate (in percent) of correctly detected number of major factors under different variations of PA when minor factors were not present under a symmetrical response distribution.

Method	Major factors		Var. per factor		Number of respondents			Number of categories		
	1	3	5	15	200	500	1000	2	3	4
PCA95KB	100	99.95	99.95	100	99.92	100	100	99.92	100	100
PCAmKB	99.89	99.92	99.86	99.95	99.71	100	100	99.71	100	100
PAFA95KB	97.33	99.86	97.61	99.59	98.25	98.71	98.84	98.3	98.21	99.29
PAFAmKB	81.17	98.81	83.92	96.06	88.88	90.63	90.46	86.46	90.59	92.92
PCA95BY	100	99.95	99.95	100	99.92	100	100	99.92	100	100
PCAmBY	99.89	99.92	99.86	99.95	99.71	100	100	99.71	100	100
PAFA95BY	97.33	99.86	97.61	99.59	98.25	98.71	98.84	98.3	98.21	99.29
PAFAmBY	81.17	98.84	83.92	96.09	88.92	90.63	90.46	86.5	90.59	92.92
PCA95Pear	100	99.97	99.97	100	99.96	100	100	99.96	100	100
PCAmPear	99.86	99.89	99.86	99.89	99.63	100	100	99.67	99.96	100
PAFA95Pear	88.73	99.81	89.17	99.36	86.5	98.21	98.09	93.04	94.46	95.3
PAFAmPear	74.45	98.53	79.31	93.67	85.13	86.92	87.42	80.09	88.13	91.25
% Ind. Matrices	0	8.12	0	8.12	11.33	0.84	0	9.17	3	0

*Note*. PCA = Principal Component Analysis, PAFA = Principal Axes Factor Analysis; 95 = 95% eigenvalue, m = mean eigenvalue; KB = polychoric correlation with Knol-Berger smoothing algorithm, BY = polychoric correlation Bentler-Yuan smoothing algorithm, Pear = Pearson; Var. per factor = Variables per major factor; % Ind. Matrices = Percentage of indefinite correlation matrices

Table 2 presents the analogous results of the simulations with symmetrical response distribution, with minor factors present.

**Table 2:** Rate (in percent) of correctly detected number of major factors under different variations of PA when major and minor factors were present under a symmetrical response distribution.

Method	Major factors		Var. per factor		Number of respondents			Number of categories		
	1	3	5	15	200	500	1000	2	3	4
PCA95KB	99.97	99.97	99.95	100	99.92	100	100	99.96	100	99.96
PCAmKB	99.95	99.89	99.84	100	99.75	100	100	99.79	100	99.96
PAFA95KB	96.67	99.86	97.2	99.33	97.63	99.08	98.09	97.67	98.55	98.58
PAFAmKB	74.45	98.89	78.75	94.59	85.92	88.5	85.59	85.09	86.75	88.17
PCA95BY	99.97	99.97	99.95	100	99.92	100	100	99.96	100	99.96
PCAmBY	99.95	99.89	99.84	100	99.75	100	100	99.79	100	99.96
PAFA95BY	96.67	99.86	97.2	99.33	97.63	99.08	98.09	97.67	98.55	98.58
PAFAmBY	74.45	98.89	78.75	94.59	85.92	88.5	85.59	85.09	86.75	88.17
PCA95Pear	99.97	100	99.97	100	99.96	100	100	100	100	99.96
PCAmPear	99.95	99.89	99.84	100	99.75	100	100	99.79	100	99.96
PAFA95Pear	88.31	99.86	89.39	98.78	88.55	97.63	96.08	91.09	95.25	95.92
PAFAmPear	66.5	98.47	74.81	90.17	82.71	85.13	79.63	77.96	84.17	85.33
% Ind. Matrices	0	8.84	0.06	8.78	11.58	1.67	0	10.08	3.17	0

*Note*. PCA = Principal Component Analysis, PAFA = Principal Axes Factor Analysis; 95 = 95% eigenvalue, m = mean eigenvalue; KB = polychoric correlation with Knol-Berger smoothing algorithm, BY = polychoric correlation Bentler-Yuan smoothing algorithm, Pear = Pearson; Var. per factor = Variables per major factor; % Ind. Matrices = Percentage of indefinite correlation matrices

The Tables 3 and 4 present the results with skewed response distribution without (Table 3) and with minor factors (Table 4).

**Table 3:** Rate (in percent) of correctly detected number of major factors under different variations of PA when only major factors were present under a skewed response distribution.

Method	Major factors		Var. per factor		Number of respondents			Number of categories		
	1	3	5	15	200	500	1000	2	3	4
PCA95KB	99.72	96.45	98.56	97.61	95.04	99.21	100	95.21	99.04	100
PCAmKB	99.03	93	96.78	95.25	90.38	97.79	99.88	91.29	96.75	100
PAFA95KB	85.11	91	88.67	87.45	84	88.25	91.92	77.8	89.34	97.04
PAFAmKB	54.75	83.42	65.97	72.2	62.67	69.46	75.13	53.83	68.67	84.75
PCA95BY	99.72	98.14	98.58	99.28	97.25	99.54	100	97.34	99.46	100
PCAmBY	99.03	95.72	96.86	97.89	93.88	98.38	99.88	94.5	97.63	100
PAFA95BY	85.11	93.7	88.7	90.11	87.13	89.17	91.92	81.13	90.04	97.04
PAFAmBY	54.81	86.78	66.03	75.56	67.08	70.17	75.13	57.88	69.67	84.84
PCA95Pear	99.92	99.25	99.53	99.64	98.84	99.92	100	98.75	100	100
PCAmPear	99.28	97.92	98.47	98.72	96.09	99.71	100	96.46	99.38	99.96
PAFA95Pear	76.5	98.19	81	93.7	74.71	92.46	94.88	81.29	87.83	92.92
PAFAmPear	51.39	93.2	68.31	76.28	67.09	73.92	75.88	60.09	73.17	83.63
% Ind. Matrices	1.11	23.14	3.06	21.2	27.04	8.58	0.75	23.67	8.42	4.29

*Note*. PCA = Principal Component Analysis, PAFA = Principal Axes Factor Analysis; 95 = 95% eigenvalue, m = mean eigenvalue; KB = polychoric correlation with Knol-Berger smoothing algorithm, BY = polychoric correlation Bentler-Yuan smoothing algorithm, Pear = Pearson; Var. per factor = Variables per major factor; % Ind. Matrices = Percentage of indefinite correlation matrices

**Table 4:** Rate (in percent) of correctly detected number of major factors under different variations of PA when major and minor factors were present under a skewed response distribution.

Method	Major factors		Var. per factor		Number of respondents			Number of categories		
	1	3	5	15	200	500	1000	2	3	4
PCA95KB	99.64	95.06	98.22	96.48	93.25	98.79	100	95.67	98.71	97.67
PCAmKB	98.75	89.34	95.78	92.31	85.46	96.71	99.96	90.46	96.75	94.92
PAFA95KB	76.89	85.19	83.95	78.14	75.42	81.63	86.08	78.59	85.54	79
PAFAmKB	37.86	72.56	56.95	53.47	48.3	56.75	60.59	50.29	61.92	53.42
PCA95BY	99.67	97.36	98.28	98.75	96.25	99.29	100	97.67	99.38	98.5
PCAmBY	98.75	93.14	95.78	96.11	90.13	97.75	99.96	94	97.79	96.04
PAFA95BY	76.98	88.95	84	81.92	80.13	82.67	86.08	81.92	86.71	80.25
PAFAmBY	37.95	76.09	57	57.03	53.17	57.29	60.59	54	62.92	54.13
PCA95Pear	99.86	99.14	99.5	99.5	98.54	99.96	100	98.88	99.88	99.75
PCAmPear	99.14	97.42	98.28	98.28	95	99.84	100	96.5	99.42	98.92
PAFA95Pear	72.03	97.5	81.14	88.39	76.79	89.5	88	80.09	86.84	87.38
PAFAmPear	37.14	91.28	63.5	64.92	60.54	67	65.09	58.54	67.04	67.04
% Ind. Matrices	0.92	26.06	2.39	24.58	29.96	8.92	1.59	23.21	8.75	8.5

*Note*. PCA = Principal Component Analysis, PAFA = Principal Axes Factor Analysis; 95 = 95% eigenvalue, m = mean eigenvalue; KB = polychoric correlation with Knol-Berger smoothing algorithm, BY = polychoric correlation Bentler-Yuan smoothing algorithm, Pear = Pearson; Var. per factor = Variables per major factor; % Ind. Matrices = Percentage of indefinite correlation matrices

### Indefinite correlation matrices

Overall, indefinite correlation matrices were observed in 8.1% of all simulated datasets. As can be seen in Tables 1 to 4, indefinite polychoric correlation matrices were generally observed more often in datasets with a large number of variables, a large number of factors and a low number of respondents. Polychoric correlation matrices also tended to be indefinite more often when the response distribution was skewed, compared to conditions when the response distribution was symmetrical, and when the number of response categories was low, compared with conditions with a higher number of response categories. In general, fewer indefinite correlation matrices were observed when factor loadings were lower.

### Overall effects of study characteristics on the accuracy of dimensionality assessment

Independent of the approach used in dimensionality assessment, some general statements can be made with regard to which conditions allowed a higher accuracy in the detection of the major factors in a dataset. In general, the number of major factors could be detected more accurately when the number of respondents was large, the response distribution was symmetrical, the number of response categories was large, when the factor loadings were high, and when no minor factors were present in the dataset.

### Accuracy of approaches based on polychoric correlation matrices and of approaches based on Pearson correlations

In datasets where indefinite polychoric correlation matrices were observed, the accuracy of methods based on Pearson correlations could be compared against methods based on the three smoothing algorithms. Compared to methods based on polychoric correlation matrices which applied the smoothing algorithm of Bentler and Yuan [42], which led to the most accurate dimensionality assessments in our simulation study, methods based on Pearson correlations were slightly less accurate. For both methods, 1152 dimensionality assessments were obtained, resulting from the four used variations of PA which were applied under 288 simulated conditions described in the method section. In these dimensionality assessments, the approach based on polychoric correlation matrices led to more accurate results in 213 cases, to less accurate results in 190 cases, and to equally accurate results in 749 cases. In general, methods based on Pearson correlations led to more accurate results only in datasets with medium factor loadings, no minor factors and a skewed response distribution, when the number of respondents was small.

Of all methods investigated in this study, the application of the modified PA of Glorfeld to a PCA of Pearson correlation and to a PCA of polychoric correlation matrices which were smoothed used the smoothing algorithm of Bentler and Yuan were the most accurate over all conditions simulated in this study. While the application of parallel analysis to a PCA of Pearson correlations led to an accurate assessment in 99.76% of all datasets, the application of PCA to polychoric correlations which were smoothed using the algorithm of Bentler and Yuan led to an accurate assessment in 99.34% of all datasets.

### Comparison of the accuracy of parallel analysis based on PCA and parallel analysis based on PAFA

Over all conditions, the combination of parallel analysis and PCA was more accurate in the determination of the number of major factors than the modified parallel analysis which was used with PAFA. The specific results depended on the type of correlation matrix and, in the case of polychoric correlation matrices, the smoothing algorithm used in the analysis. For PCA of polychoric correlation matrices with smoothing algorithms of Knol and Berger [31] or Bentler and Yuan [42], the accuracy of the dimensionality assessment tended to be near 100% under all conditions. The parallel analysis with PAFA, when applied to polychoric correlation matrices with or without smoothing algorithms, was less accurate. Under almost all conditions, variations of parallel analysis which used the criterion of Glorfeld [7] were more accurate then approaches which used the original criterion of Horn [8]. Parallel analysis with PCA of Pearson correlation matrices led in general to an accurate assessment of the dimensionality of a dataset, while parallel analysis with PAFA of Pearson correlation matrices was less accurate than when applied to polychoric correlation matrices when the response distribution was symmetrical, or skewed with high factor loadings, but more accurate when the response distribution was skewed with medium factor loadings.

## Discussion

The use of polychoric and tetrachoric correlation coefficients in the application of PA and PCA has long been considered as an important alternative to approaches based on Pearson correlation coefficients. Timmerman and Lorenzo-Seva [2] compared both approaches, but found that the calculation of the polychoric correlation matrix often was not possible due to the non-convergence of the estimation procedure of Olsson [30] or led to non-positive definite correlation matrices. In this paper, we investigated the application of the smoothing algorithms described by Knol and Berger [31], Higham [40] and Bentler and Yuan [42], which are available via R software packages, to circumvent the problem of indefinite correlation matrices. In line with previous studies, we observed indefinite correlation matrices more often when the number of simulated respondents was small [16, 26], when the number of simulated variables was large [26], when the factor loadings were high [15,26], and when the distribution of the responses was skewed over the response categories.

Like Timmerman and Lorenzo-Seva [2], we observed in a preliminary study that the two-step estimation procedure by Olsson [30] did not always converge. For this reason, we applied a variation of the two-step estimation procedure of Olsson [30] in our simulation study, which used an item-pair specific estimation of the threshold parameters for the estimation of the polychoric correlation coefficient. It should be noted that this approach deviates from the commonly utilized model underlying the calculation of the polychoric correlation coefficient, which assumes that the item specific threshold are invariant over all item pairs. Therefore, we do not recommend the application of this algorithm especially in large datasets; in small datasets, which were also simulated as part of our simulation study, the application of this procedure seems to be necessary to avoid convergence problems which occur if threshold parameters which are invariant over the item pairs are calculated as part of the estimation of the polychoric correlation coefficient. In our study, the application of this adapted estimation procedure and the smoothing algorithms of Knol and Berger [31], Higham [40] or Bentler and Yuan [42] allowed the application of PCA and PAFA under all simulated conditions. Although we are not aware of any studies which compared the variation of the two-step estimation procedure of Olsson [30], which was used in our study, with an approach that estimates threshold parameters which are invariant for each item over all item pairs, both approaches are expected to lead to similar results if the model underlying the calculation of the polychoric correlation coefficient fits the data well. It can therefore be assumed that the results of our simulation study only depend to a small degree on the choice of the estimation method for the polychoric correlations. Based on the results of our study, the variation of the two-step procedure for calculating the polychoric correlation coefficient which was used in our study can be recommended if convergence problems with either the two-step procedure or the maximum likelihood approach occur. If no convergence problems occur, approaches which use invariant threshold parameters for all item pairs may be preferable from a theoretical perspective. The maximum likelihood approach and the two-step procedure for calculating the polychoric correlation coefficient were discussed by Olsson [30], who argued that both approaches should be comparable from a practical perspective. A major result from our simulation study seems to be that PA variations based on PAFA and PCA seem to differ with regard to their accuracy as measures of the number of major factors present in a dataset, which we used as a measure of dimensionality. A similar result was reported by Timmerman and Lorenzo-Seva [2]. Garrido et al. [16] also stated that the application of PA is not suited for EFA on theoretical reasons. This result is in our view of major practical importance, since PCA is mainly used as a data reduction technique, while EFA is based on the common factor model (for a more detailed discussion of both approaches, see [1], pp. 274–276). Although PA combined with PCA seems to lead to more accurate dimensionality assessment, it should be pointed out that PCA is not based on a common factor model, and therefore an additional application of exploratory factor analysis could be considered, if this model is found to be appropriate.

Recent simulation studies [2,16] have recommended the use of PA and MRFA as a method of EFA to determine the dimensionality of an item set. To the best of our knowledge, this method is currently implemented in the Factor software [48], but not available in any published R package. Since it is not clear if an implementation of this method in R would lead to comparable results as the Factor software, we did not investigate this method in our simulation study. However, the simulation study of Garrido et al. [16] investigated the accuracy of PA and MRFA in categorical data while using the smoothing algorithm of Knol and Berger [31] if indefinite polychoric correlation matrices were observed. Based on these results, it can be concluded that PA based on MRFA leads to accurate results when applied to smoothed polychoric or tetrachoric correlation matrices if the smoothing algorithm of Knol and Berger [31] is used. Therefore, the use of PA and MRFA can be recommended for practical application based on the results reported so far in the literature, especially if the latent factors underlying a dataset can be expected to be uncorrelated and if the number of variables per factor is large. However, future studies should investigate which effect the choice of smoothing algorithm has on the accuracy of the analysis when PA is applied to MRFA.

We observed that all methods tended to estimate the number of underlying dimensions to be higher than the number of major factors contained in the data when minor factors were present in the data and when the number of respondents increased. This result was to be expected, since the presence of minor factors influences the dimensionality of an item set. However, this result is relevant for the practical application of PA and PCA of smoothed polychoric and Pearson correlation matrices as a measure of dimensionality, since minor factors may be present in realistic datasets [49]. Under these conditions, the use of additional measures of dimensionality may be advisable to assess the practical influence of each obtained dimension for modeling the observed data. A recent paper [50] described an approach based on a numerical convex hull-based heuristic, which may be appropriate for this problem.

It has already been noted that over all conditions and methods simulated in our study, choosing the 95^th^ percentile of the random eigenvalue distribution seems to lead to a more accurate assessment of the number of major factors, especially when larger item sets were examined. This observation has already been reported in a similar simulation study [2], but seems to contradict the recommendations of Garrido et al. [16], who found that using the mean of the random eigenvalue distribution in a PCA of polychoric correlations led to superior results. As Garrido et al. [16] noted, the use of the mean as a summary statistic for parallel analysis led to superior results in conditions with correlated factors, few variables per factor, and small sample sizes of 100 respondents. Since these conditions were only partly met in our simulations, our results thus do not contradict the results reported by Garrido et al. [16]. Results of Garrido et al. [16] and our own results concurrently indicate that the effect of the number of variables per factor on the accuracy of PA may interact with other effects, such as the effect of correlations between factors.

A main conclusion of our simulation study pertains to the practical differences between the smoothing algorithms of Bentler and Yuan [42], Higham [40] and Knol and Berger [31]. While the algorithm of Higham [40] is based on the idea of minimizing the distance, defined by the Frobenius norm, between the observed correlation matrix and the smoothed correlation matrix, the algorithm of Bentler and Yuan [42] is based on the idea of rescaling specific rows and columns of the correlation matrix. The algorithm of Knol and Berger [31] is based on the idea of retaining the original eigenvalues while replacing eigenvalues below a predefined constant with this constant. Of all algorithms, the approach of Bentler and Yuan [42] is associated with the fewest changes to the correlation matrix and may thus be considered to be preferable from a theoretical point of view. In our simulation study, methods based on the algorithms of Knol and Berger [31], Higham [40], and Bentler and Yuan [42] showed a comparable performance with regard to the accuracy to detect the number of underlying major factors, with a slightly better performance of methods based on the Bentler andYuan [42] algorithm. We therefore recommend the application of the algorithm of Bentler and Yuan [42] for the assessment of dimensionality.

When compared with methods based on Pearson correlations, we found methods based on smoothed polychoric correlation matrices to be of comparable accuracy for dimensionality assessment, especially if the smoothing algorithm of Bentler and Yuan [42] was applied. Moreover, it should be noted that the application of the modified parallel analysis of Glorfeld to a PCA of Pearson correlation was among the most accurate methods in this study. Similar findings have been reported by Cho et al. [33] and Timmerman and Lorenzo-Seva [2].

However, we observed that the differences in the accuracy of the various methods based on Pearson correlations and on polychoric correlations depended on the characteristics of the analyzed datasets. In datasets with skewed response distributions, high factor loadings and a sufficiently large sample of respondents, the differences between methods based on Pearson correlations and methods based on polychoric correlations were especially pronounced. These findings are in line with recommendations by Garrido et al. [16]. Furthermore, it should be noted that methods based on smoothed polychoric correlation matrices seem to be more accurate than methods based on the analysis of indefinite polychoric correlation matrices. Timmerman and Lorenzo-Seva [2] noted that they experienced convergence problems with their smoothing algorithm. In their study, a smoothing algorithm described by [50] was used (Lorenzo-Seva, personal communication). We did not experience similar problems in any of our simulated datasets, although these problems could occur in applied research. Based on the results found in our study, convergence problems could be avoided if polychoric correlations are estimated using the variation of the two-step procedure of Olsson [30], which was used in our study.

In datasets with medium factor loadings, a skewed response distribution and a small sample of respondents, PA of Pearson correlations tended to be more accurate than PA based on polychoric correlations. This finding may be surprising given the results reported in a previous simulation study [16], which found that a PCA of polychoric correlations is more accurate than a PCA of Pearson correlations in datasets with a skewed response distribution. A close examination of our results shows that under these conditions, the critical eigenvalues reported by parallel analysis are very close to each other. On the other hand, the variance of the expected eigenvalues is larger for the polychoric correlation matrices in small samples than for the Pearson correlation matrices. It follows that the dimensionality assessment of PA with PCA or EFA of polychoric correlations is thus more dependent on the characteristics of the analyzed sample than with methods based on Pearson correlations under these conditions.

Overall, our study indicates that under the simulated conditions, PA and PCA of smoothed polychoric correlation matrices may serve as a good indicator of the dimensionality of an item set. Also a PCA based on Pearson correlations which used the Glorfield criterion for determining the critical values was found to lead to accurate results under the conditions simulated in our study. PAFA and other methods based on the analysis of Pearson correlation matrices may be less accurate under specific conditions. However, in the presence of minor factors, this method may over-estimate the dimensionality of an item set as measured by the number of present major factors. Therefore, we advise against the use of PA with PAFA extraction as a measure of dimensionality under conditions similar to those simulated in our study in practical research.

There are several limitations to the generalizability of our study. First, the data which were analyzed in the simulation study were constructed according to a specific rationale which resembles those used in previous simulation studies [2,16] and were aimed to resemble datasets used in practical research. Empirical datasets may differ from the conditions simulated in our study with regard to several characteristics which influence the accuracy of PA, like the underlying factor structure. Moreover, our data construction was based on a common factor model, which may not be the appropriate model in practical measurements. Furthermore, the simulated datasets were based on a simple structure model with uncorrelated factors, in which parallel analysis has been shown to perform better than in datasets with correlated factors [16]. In real datasets, significant correlations between latent factors may be observed. It should be noted that the presence of correlated factors might also affect the accuracy of methods based on parallel analysis which are applied to smoothed polychoric correlation matrices, and that their effect on accuracy may depend on the chosen smoothing algorithm. It follows that the conditions simulated in our study should be considered as an idealized scenario, and do not measure the robustness of PA under specific conditions which include non-linearity.

Second, the methods used for dimensionality assessment were restricted to PCA and PAFA, two methods which were also investigated in several recent studies [2,32,34]. However, this is to the best of our knowledge the first study which compared the effect of different smoothing algorithms on the accuracy of these methods in the presence of minor factors. Several variations have been suggested to these methods, which may be less common, but may be more accurate than those used in our study under specific conditions [2].

However, despite these limitations, our study allows some recommendations for the applied researcher who wishes to use PA to determine the dimensionality of a set of ordinal variables: First, the application of PCA of Pearson correlations or smoothed polychoric correlations seems to be more accurate to determine the number of major factors underlying a set of ordinal variables than PAFA. This difference seems to be more pronounced in the presence of minor factors, which may be the case in many real datasets, and if the response distribution is skewed. Second, under the conditions simulated in our study, the modified PA of Glorfeld [7] seems to lead to a more accurate assessment of dimensionality than the original approach of Horn [8]. This effect is more pronounced when PA is used in the context of EFA. Third, under certain conditions the choice of an appropriate smoothing algorithm can affect the accuracy of dimensionality assessments based on PA. Of the smoothing algorithms used in our study, the algorithm described by Bentler and Yuan [42] seems to lead to more accurate results when used with PA based on PCA and can therefore be recommended.

## Supporting Information

S1 Appendix Detailed results for the accuracy of parallel analysis in datasets with high and medium factor loadings. (DOCX)
