# Supplementary material for: Comparing the Effects of Different Smoothing Algorithms on the Assessment of Dimensionality of Ordered Categorical Items with Parallel Analysis
Source: PLoS One. 2016 Feb 4;11(2):e0148143. doi: 10.1371/journal.pone.0148143 (PMC4742070; doi:10.1371/journal.pone.0148143)
Supplement: S1 Appendix — (DOCX) [file pone.0148143.s001.docx]

**Supplementary materials**

In this supplementary section, we provide additional detailed results for the accuracy of parallel analysis in datasets with high and medium factor loadings. As in the main text, each dimensionality assessment was considered as accurate if the number of major factors was correctly measured. The following tables present the percentage of simulated datasets in which the number of major factors was reported by the application of parallel analysis. In contrast to the main text, the entries in the individual cells of these tables are only based on 1800 datasets (in columns which present results for different numbers of major factors or different numbers of variables per factor) or 1200 datasets (in columns which present results for different numbers of respondents or different response categories). It follows that the confidence intervals for the individual entries in these tables are smaller than ± 2.3 for entries based on 1800 datasets and smaller than ± 2.8 for entries based on 1200 datasets, based on a confidence level of 0.95. However, it should be noted that the size of the confidence interval is smaller for large and small entries. For entries above 90, the respective confidence intervals are smaller than ± 1.4 for entries based on 1800 datasets and smaller than ± 1.7 for entries based on 1200 datasets, again based on a significance level of 0.95.

**Detailed results for datasets with high factor loadings**

Table S1 summarizes the results of the simulations where the response distribution was symmetrical and the factor loadings were high, with no minor factors present.

Table S1. Rate (in percent) of correctly detected number of major factors under different variations of PA when minor factors were not present under a symmetrical response distribution in datasets with high factor loadings

| Method | Major factors | | Var. per factor | | Number of respondents | | | Number of categories categories | | |
| --- | --- | --- | --- | --- | --- | --- | --- | --- | --- | --- |
|  | 1 | 3 | 5 | 15 | 200 | 500 | 1000 | 2 | 3 | 4 |
| PCA95KB | 100 | 100 | 100 | 100 | 100 | 100 | 100 | 100 | 100 | 100 |
| PCAmKB | 100 | 100 | 100 | 100 | 100 | 100 | 100 | 100 | 100 | 100 |
| PAFA95KB | 99.44 | 100 | 99.44 | 100 | 99.67 | 99.83 | 99.67 | 99.67 | 99.75 | 99.75 |
| PAFAmKB | 90.94 | 100 | 90.94 | 100 | 96 | 94.92 | 95.5 | 92.67 | 95.92 | 97.83 |
| PCA95BY | 100 | 100 | 100 | 100 | 100 | 100 | 100 | 100 | 100 | 100 |
| PCAmBY | 100 | 100 | 100 | 100 | 100 | 100 | 100 | 100 | 100 | 100 |
| PAFA95BY | 99.44 | 100 | 99.44 | 100 | 99.67 | 99.83 | 99.67 | 99.67 | 99.75 | 99.75 |
| PAFAmBY | 90.94 | 100 | 90.94 | 100 | 96 | 94.92 | 95.5 | 92.67 | 95.92 | 97.83 |
| PCA95Pear | 100 | 100 | 100 | 100 | 100 | 100 | 100 | 100 | 100 | 100 |
| PCAmPear | 100 | 100 | 100 | 100 | 100 | 100 | 100 | 100 | 100 | 100 |
| PAFA95Pear | 97.67 | 100 | 97.67 | 100 | 98.5 | 99.33 | 98.67 | 97.33 | 99.75 | 99.42 |
| PAFAmPear | 83.33 | 100 | 85.33 | 98 | 91.42 | 91.42 | 92.17 | 85.67 | 93.58 | 95.75 |
| % Ind. Matrices | 0 | 10.67 | 0 | 10.67 | 14.33 | 1.67 | 0 | 10 | 6 | 0 |

*Note.* PCA = Principal Component Analysis, PAFA = Principal Axes Factor Analysis; 95 = 95% eigenvalue, m = mean eigenvalue; KB = polychoric correlation with Knol-Berger smoothing algorithm, BY = polychoric correlation Bentler-Yuan smoothing algorithm, Pear = Pearson; Var. per factor = Variables per major factor; % Ind. Matrices = Percentage of indefinite correlation matrices

Table S2 presents the analogous results of the simulations of datasets with high factor loadings with symmetrical response distribution, with minor factors present.

Table S2. Rate (in percent) of correctly detected number of major factors under different variations of PA when major and minor factors were present under a symmetrical response distribution in datasets with high factor loadings

| Method | Major factors | | Var. per factor | | Number of respondents | | | Number of categories categories | | |
| --- | --- | --- | --- | --- | --- | --- | --- | --- | --- | --- |
|  | 1 | 3 | 5 | 15 | 200 | 500 | 1000 | 2 | 3 | 4 |
| PCA95KB | 100 | 100 | 100 | 100 | 100 | 100 | 100 | 100 | 100 | 100 |
| PCAmKB | 100 | 100 | 100 | 100 | 100 | 100 | 100 | 100 | 100 | 100 |
| PAFA95KB | 99.06 | 100 | 99.11 | 99.94 | 99.75 | 99.58 | 99.25 | 99.33 | 99.42 | 99.83 |
| PAFAmKB | 82.89 | 100 | 84.89 | 98 | 92.58 | 92.5 | 89.25 | 90.67 | 91.33 | 92.33 |
| PCA95BY | 100 | 100 | 100 | 100 | 100 | 100 | 100 | 100 | 100 | 100 |
| PCAmBY | 100 | 100 | 100 | 100 | 100 | 100 | 100 | 100 | 100 | 100 |
| PAFA95BY | 99.06 | 100 | 99.11 | 99.94 | 99.75 | 99.58 | 99.25 | 99.33 | 99.42 | 99.83 |
| PAFAmBY | 82.89 | 100 | 84.89 | 98 | 92.58 | 92.5 | 89.25 | 90.67 | 91.33 | 92.33 |
| PCA95Pear | 100 | 100 | 100 | 100 | 100 | 100 | 100 | 100 | 100 | 100 |
| PCAmPear | 100 | 100 | 100 | 100 | 100 | 100 | 100 | 100 | 100 | 100 |
| PAFA95Pear | 94.94 | 100 | 96 | 98.94 | 98.17 | 98.67 | 95.58 | 95.17 | 98.58 | 98.67 |
| PAFAmPear | 72.56 | 100 | 78.89 | 93.67 | 89.08 | 89.42 | 80.33 | 81.83 | 88.17 | 88.83 |
| % Ind. Matrices | 0 | 12.11 | 0.11 | 12 | 14.83 | 3.33 | 0 | 11.83 | 6.33 | 0 |

*Note.* PCA = Principal Component Analysis, PAFA = Principal Axes Factor Analysis; 95 = 95% eigenvalue, m = mean eigenvalue; KB = polychoric correlation with Knol-Berger smoothing algorithm, BY = polychoric correlation Bentler-Yuan smoothing algorithm, Pear = Pearson; Var. per factor = Variables per major factor; % Ind. Matrices = Percentage of indefinite correlation matrices

The Tables S3 and S4 present the results with skewed response distribution without (Table S3) and with minor factors (Table S4).

Table S3. Rate (in percent) of correctly detected number of major factors under different variations of PA when only major factors were present under a skewed response distribution in datasets with high factor loadings

| Method | Major factors | | Var. per factor | | Number of respondents | | | Number of categories categories | | |
| --- | --- | --- | --- | --- | --- | --- | --- | --- | --- | --- |
|  | 1 | 3 | 5 | 15 | 200 | 500 | 1000 | 2 | 3 | 4 |
| PCA95KB | 100 | 100 | 100 | 100 | 100 | 100 | 100 | 100 | 100 | 100 |
| PCAmKB | 100 | 99.89 | 99.94 | 99.94 | 99.83 | 100 | 100 | 99.83 | 100 | 100 |
| PAFA95KB | 93.89 | 99.72 | 95 | 98.61 | 95.67 | 96.75 | 98 | 95.67 | 98.25 | 96.5 |
| PAFAmKB | 67.72 | 98.5 | 75.94 | 90.28 | 80.75 | 83.58 | 85 | 79.08 | 86.42 | 83.83 |
| PCA95BY | 100 | 99.94 | 99.94 | 100 | 99.92 | 100 | 100 | 99.92 | 100 | 100 |
| PCAmBY | 100 | 100 | 100 | 100 | 100 | 100 | 100 | 100 | 100 | 100 |
| PAFA95BY | 93.89 | 99.72 | 95 | 98.61 | 95.67 | 96.75 | 98 | 95.67 | 98.25 | 96.5 |
| PAFAmBY | 67.83 | 99 | 76.06 | 90.78 | 81.58 | 83.67 | 85 | 79.83 | 86.42 | 84 |
| PCA95Pear | 100 | 100 | 100 | 100 | 100 | 100 | 100 | 100 | 100 | 100 |
| PCAmPear | 100 | 99.94 | 100 | 99.94 | 99.92 | 100 | 100 | 99.92 | 100 | 100 |
| PAFA95Pear | 84.33 | 99.94 | 87 | 97.28 | 82.92 | 97 | 96.5 | 88.58 | 92.58 | 95.25 |
| PAFAmPear | 56.72 | 99.33 | 74 | 82.06 | 76.17 | 79.08 | 78.83 | 70.5 | 81.58 | 82 |
| % Ind. Matrices | 1.89 | 28 | 4.5 | 25.39 | 34.5 | 8.83 | 1.5 | 27.75 | 8.5 | 8.58 |

*Note.* PCA = Principal Component Analysis, PAFA = Principal Axes Factor Analysis; 95 = 95% eigenvalue, m = mean eigenvalue; KB = polychoric correlation with Knol-Berger smoothing algorithm, BY = polychoric correlation Bentler-Yuan smoothing algorithm, Pear = Pearson; Var. per factor = Variables per major factor; % Ind. Matrices = Percentage of indefinite correlation matrices

Table S4. Rate (in percent) of correctly detected number of major factors under different variations of PA when major and minor factors were present under a skewed response distribution in datasets with high factor loadings

| Method | Major factors | | Var. per factor | | Number of respondents | | | Number of categories | | |
| --- | --- | --- | --- | --- | --- | --- | --- | --- | --- | --- |
|  | 1 | 3 | 5 | 15 | 200 | 500 | 1000 | 2 | 3 | 4 |
| PCA95KB | 100 | 99.83 | 99.94 | 99.89 | 99.75 | 100 | 100 | 99.75 | 100 | 100 |
| PCAmKB | 100 | 99.67 | 99.78 | 99.89 | 99.5 | 100 | 100 | 99.5 | 100 | 100 |
| PAFA95KB | 91.22 | 98.94 | 93.72 | 96.44 | 93.75 | 96.42 | 95.08 | 94.67 | 96.58 | 94 |
| PAFAmKB | 55.83 | 97.06 | 71.89 | 81 | 76.17 | 79.5 | 73.67 | 75 | 80 | 74.33 |
| PCA95BY | 100 | 99.94 | 99.94 | 100 | 99.92 | 100 | 100 | 99.92 | 100 | 100 |
| PCAmBY | 100 | 99.83 | 99.83 | 100 | 99.75 | 100 | 100 | 99.75 | 100 | 100 |
| PAFA95BY | 91.28 | 99.33 | 93.83 | 96.78 | 94.42 | 96.42 | 95.08 | 95.17 | 96.58 | 94.17 |
| PAFAmBY | 56 | 97.56 | 71.94 | 81.61 | 77.08 | 79.58 | 73.67 | 75.92 | 80 | 74.42 |
| PCA95Pear | 100 | 100 | 100 | 100 | 100 | 100 | 100 | 100 | 100 | 100 |
| PCAmPear | 100 | 99.94 | 100 | 99.94 | 99.92 | 100 | 100 | 99.92 | 100 | 100 |
| PAFA95Pear | 82.11 | 99.78 | 90 | 91.89 | 91.33 | 92.67 | 88.83 | 86.42 | 92.67 | 93.75 |
| PAFAmPear | 43 | 98.39 | 68.89 | 72.5 | 72.75 | 72.33 | 67 | 65.75 | 73.08 | 73.25 |
| % Ind. Matrices | 1.33 | 28.83 | 3.72 | 26.44 | 32.58 | 9.5 | 3.17 | 27.42 | 9.17 | 8.67 |

*Note.* PCA = Principal Component Analysis, PAFA = Principal Axes Factor Analysis; 95 = 95% eigenvalue, m = mean eigenvalue; KB = polychoric correlation with Knol-Berger smoothing algorithm, BY = polychoric correlation Bentler-Yuan smoothing algorithm, Pear = Pearson; Var. per factor = Variables per major factor; % Ind. Matrices = Percentage of indefinite correlation matrices

**Detailed results for datasets with medium factor loadings**

Table S5 summarizes the results of the simulations where the response distribution was symmetrical and the factor loadings were of medium size, with no minor factors present.

Table S5. Rate (in percent) of correctly detected number of major factors under different variations of PA when minor factors were not present under a symmetrical response distribution in datasets with medium factor loadings

| Method | Major factors | | Var. per factor | | Number of respondents | | | Number of categories categories | | |
| --- | --- | --- | --- | --- | --- | --- | --- | --- | --- | --- |
|  | 1 | 3 | 5 | 15 | 200 | 500 | 1000 | 2 | 3 | 4 |
| PCA95KB | 100 | 99.89 | 99.89 | 100 | 99.83 | 100 | 100 | 99.83 | 100 | 100 |
| PCAmKB | 99.78 | 99.83 | 99.72 | 99.89 | 99.42 | 100 | 100 | 99.42 | 100 | 100 |
| PAFA95KB | 95.22 | 99.72 | 95.78 | 99.17 | 96.83 | 97.58 | 98 | 96.92 | 96.67 | 98.83 |
| PAFAmKB | 71.39 | 97.61 | 76.89 | 92.11 | 81.75 | 86.33 | 85.42 | 80.25 | 85.25 | 88 |
| PCA95BY | 100 | 99.89 | 99.89 | 100 | 99.83 | 100 | 100 | 99.83 | 100 | 100 |
| PCAmBY | 99.78 | 99.83 | 99.72 | 99.89 | 99.42 | 100 | 100 | 99.42 | 100 | 100 |
| PAFA95BY | 95.22 | 99.72 | 95.78 | 99.17 | 96.83 | 97.58 | 98 | 96.92 | 96.67 | 98.83 |
| PAFAmBY | 71.39 | 97.67 | 76.89 | 92.17 | 81.83 | 86.33 | 85.42 | 80.33 | 85.25 | 88 |
| PCA95Pear | 100 | 99.94 | 99.94 | 100 | 99.92 | 100 | 100 | 99.92 | 100 | 100 |
| PCAmPear | 99.72 | 99.78 | 99.72 | 99.78 | 99.25 | 100 | 100 | 99.33 | 99.92 | 100 |
| PAFA95Pear | 79.78 | 99.61 | 80.67 | 98.72 | 74.5 | 97.08 | 97.5 | 88.75 | 89.17 | 91.17 |
| PAFAmPear | 65.56 | 97.06 | 73.28 | 89.33 | 78.83 | 82.42 | 82.67 | 74.5 | 82.67 | 86.75 |
| % Ind. Matrices | 0 | 5.56 | 0 | 5.56 | 8.33 | 0 | 0 | 8.33 | 0 | 0 |

*Note.* PCA = Principal Component Analysis, PAFA = Principal Axes Factor Analysis; 95 = 95% eigenvalue, m = mean eigenvalue; KB = polychoric correlation with Knol-Berger smoothing algorithm, BY = polychoric correlation Bentler-Yuan smoothing algorithm, Pear = Pearson; Var. per factor = Variables per major factor; % Ind. Matrices = Percentage of indefinite correlation matrices

Table S6 presents the results of the simulations of datasets with medium factor loadings with symmetrical response distribution, with minor factors present.

Table S6. Rate (in percent) of correctly detected number of major factors under different variations of PA when major and minor factors were present under a symmetrical response distribution in datasets with medium factor loadings

| Method | Major factors | | Var. per factor | | Number of respondents | | | Number of categories categories | | |
| --- | --- | --- | --- | --- | --- | --- | --- | --- | --- | --- |
|  | 1 | 3 | 5 | 15 | 200 | 500 | 1000 | 2 | 3 | 4 |
| PCA95KB | 99.94 | 99.94 | 99.89 | 100 | 99.83 | 100 | 100 | 99.92 | 100 | 99.92 |
| PCAmKB | 99.89 | 99.78 | 99.67 | 100 | 99.5 | 100 | 100 | 99.58 | 100 | 99.92 |
| PAFA95KB | 94.28 | 99.72 | 95.28 | 98.72 | 95.5 | 98.58 | 96.92 | 96 | 97.67 | 97.33 |
| PAFAmKB | 66 | 97.78 | 72.61 | 91.17 | 79.25 | 84.5 | 81.92 | 79.5 | 82.17 | 84 |
| PCA95BY | 99.94 | 99.94 | 99.89 | 100 | 99.83 | 100 | 100 | 99.92 | 100 | 99.92 |
| PCAmBY | 99.89 | 99.78 | 99.67 | 100 | 99.5 | 100 | 100 | 99.58 | 100 | 99.92 |
| PAFA95BY | 94.28 | 99.72 | 95.28 | 98.72 | 95.5 | 98.58 | 96.92 | 96 | 97.67 | 97.33 |
| PAFAmBY | 66 | 97.78 | 72.61 | 91.17 | 79.25 | 84.5 | 81.92 | 79.5 | 82.17 | 84 |
| PCA95Pear | 99.94 | 100 | 99.94 | 100 | 99.92 | 100 | 100 | 100 | 100 | 99.92 |
| PCAmPear | 99.89 | 99.78 | 99.67 | 100 | 99.5 | 100 | 100 | 99.58 | 100 | 99.92 |
| PAFA95Pear | 81.67 | 99.72 | 82.78 | 98.61 | 78.92 | 96.58 | 96.58 | 87 | 91.92 | 93.17 |
| PAFAmPear | 60.44 | 96.94 | 70.72 | 86.67 | 76.33 | 80.83 | 78.92 | 74.08 | 80.17 | 81.83 |
| % Ind. Matrices | 0 | 5.56 | 0 | 5.56 | 8.33 | 0 | 0 | 8.33 | 0 | 0 |

*Note.* PCA = Principal Component Analysis, PAFA = Principal Axes Factor Analysis; 95 = 95% eigenvalue, m = mean eigenvalue; KB = polychoric correlation with Knol-Berger smoothing algorithm, BY = polychoric correlation Bentler-Yuan smoothing algorithm, Pear = Pearson; Var. per factor = Variables per major factor; % Ind. Matrices = Percentage of indefinite correlation matrices

The Tables S7 and S8 present the results with skewed response distribution without (Table S7) and with minor factors (Table S8).

Table S7. Rate (in percent) of correctly detected number of major factors under different variations of PA when only major factors were present under a skewed response distribution in datasets with medium factor loadings

| Method | Major factors | | Var. per factor | | Number of respondents | | | Number of categories categories | | |
| --- | --- | --- | --- | --- | --- | --- | --- | --- | --- | --- |
|  | 1 | 3 | 5 | 15 | 200 | 500 | 1000 | 2 | 3 | 4 |
| PCA95KB | 99.44 | 92.89 | 97.11 | 95.22 | 90.08 | 98.42 | 100 | 90.42 | 98.08 | 100 |
| PCAmKB | 98.06 | 86.11 | 93.61 | 90.56 | 80.92 | 95.58 | 99.75 | 82.75 | 93.5 | 100 |
| PAFA95KB | 76.33 | 82.28 | 82.33 | 76.28 | 72.33 | 79.75 | 85.83 | 59.92 | 80.42 | 97.58 |
| PAFAmKB | 41.78 | 68.33 | 56 | 54.11 | 44.58 | 55.33 | 65.25 | 28.58 | 50.92 | 85.67 |
| PCA95BY | 99.44 | 96.33 | 97.22 | 98.56 | 94.58 | 99.08 | 100 | 94.75 | 98.92 | 100 |
| PCAmBY | 98.06 | 91.44 | 93.72 | 95.78 | 87.75 | 96.75 | 99.75 | 89 | 95.25 | 100 |
| PAFA95BY | 76.33 | 87.67 | 82.39 | 81.61 | 78.58 | 81.58 | 85.83 | 66.58 | 81.83 | 97.58 |
| PAFAmBY | 41.78 | 74.56 | 56 | 60.33 | 52.58 | 56.67 | 65.25 | 35.92 | 52.92 | 85.67 |
| PCA95Pear | 99.83 | 98.5 | 99.06 | 99.28 | 97.67 | 99.83 | 100 | 97.5 | 100 | 100 |
| PCAmPear | 98.56 | 95.89 | 96.94 | 97.5 | 92.25 | 99.42 | 100 | 93 | 98.75 | 99.92 |
| PAFA95Pear | 68.67 | 96.44 | 75 | 90.11 | 66.5 | 87.92 | 93.25 | 74 | 83.08 | 90.58 |
| PAFAmPear | 46.06 | 87.06 | 62.61 | 70.5 | 58 | 68.75 | 72.92 | 49.67 | 64.75 | 85.25 |
| % Ind. Matrices | 0.33 | 18.28 | 1.61 | 17 | 19.58 | 8.33 | 0 | 19.58 | 8.33 | 0 |

*Note.* PCA = Principal Component Analysis, PAFA = Principal Axes Factor Analysis; 95 = 95% eigenvalue, m = mean eigenvalue; KB = polychoric correlation with Knol-Berger smoothing algorithm, BY = polychoric correlation Bentler-Yuan smoothing algorithm, Pear = Pearson; Var. per factor = Variables per major factor; % Ind. Matrices = Percentage of indefinite correlation matrices

Table S8. Rate (in percent) of correctly detected number of major factors under different variations of PA when major and minor factors were present under a skewed response distribution in datasets with medium factor loadings

| Method | Major factors | | Var. per factor | | Number of respondents | | | Number of categories | | |
| --- | --- | --- | --- | --- | --- | --- | --- | --- | --- | --- |
|  | 1 | 3 | 5 | 15 | 200 | 500 | 1000 | 2 | 3 | 4 |
| PCA95KB | 99.28 | 90.28 | 96.5 | 93.06 | 86.75 | 97.58 | 100 | 91.58 | 97.42 | 95.33 |
| PCAmKB | 97.5 | 79 | 91.78 | 84.72 | 71.42 | 93.42 | 99.92 | 81.42 | 93.5 | 89.83 |
| PAFA95KB | 62.56 | 71.44 | 74.17 | 59.83 | 57.08 | 66.83 | 77.08 | 62.5 | 74.5 | 64 |
| PAFAmKB | 19.89 | 48.06 | 42 | 25.94 | 20.42 | 34 | 47.5 | 25.58 | 43.83 | 32.5 |
| PCA95BY | 99.33 | 94.78 | 96.61 | 97.5 | 92.58 | 98.58 | 100 | 95.42 | 98.75 | 97 |
| PCAmBY | 97.5 | 86.44 | 91.72 | 92.22 | 80.5 | 95.5 | 99.92 | 88.25 | 95.58 | 92.08 |
| PAFA95BY | 62.67 | 78.56 | 74.17 | 67.06 | 65.83 | 68.92 | 77.08 | 68.67 | 76.83 | 66.33 |
| PAFAmBY | 19.89 | 54.61 | 42.06 | 32.44 | 29.25 | 35 | 47.5 | 32.08 | 45.83 | 33.83 |
| PCA95Pear | 99.72 | 98.28 | 99 | 99 | 97.08 | 99.92 | 100 | 97.75 | 99.75 | 99.5 |
| PCAmPear | 98.28 | 94.89 | 96.56 | 96.61 | 90.08 | 99.67 | 100 | 93.08 | 98.83 | 97.83 |
| PAFA95Pear | 61.94 | 95.22 | 72.28 | 84.89 | 62.25 | 86.33 | 87.17 | 73.75 | 81 | 81 |
| PAFAmPear | 31.28 | 84.17 | 58.11 | 57.33 | 48.33 | 61.67 | 63.17 | 51.33 | 61 | 60.83 |
| % Ind. Matrices | 0.5 | 23.28 | 1.06 | 22.72 | 27.33 | 8.33 | 0 | 19 | 8.33 | 8.33 |

*Note.* PCA = Principal Component Analysis, PAFA = Principal Axes Factor Analysis; 95 = 95% eigenvalue, m = mean eigenvalue; KB = polychoric correlation with Knol-Berger smoothing algorithm, BY = polychoric correlation Bentler-Yuan smoothing algorithm, Pear = Pearson; Var. per factor = Variables per major factor; % Ind. Matrices = Percentage of indefinite correlation matrices
